# Study on the predictive value of NHR and UHR levels in early pregnancy for gestational diabetes mellitus: based on a retrospective analysis

**DOI:** 10.3389/fendo.2026.1848926

**Published:** 2026-05-29

**Authors:** Ting Yang, Yajie Duan, Changjuan Zheng

**Affiliations:** Laboratory Department of Yuncheng Central Hospital, Yuncheng, Shanxi, China

**Keywords:** gestational diabetes mellitus, neutrophil to high-density lipoprotein cholesterol ratio, predictive value, retrospective study, uric acid to high-density lipoprotein cholesterol ratio

## Abstract

**Objective:**

This study aims to examine the relationship between the neutrophil to high-density lipoprotein cholesterol ratio (NHR) and the uric acid to high-density lipoprotein cholesterol ratio (UHR) in early pregnancy and the development of GDM, and to analyze the predictive value of these factors in combination, with a view to providing a basis for the early clinical prevention and treatment of GDM.

**Methods:**

The study population comprised primiparous women carrying a single fetus who received routine antenatal care and examinations at the Obstetrics Department of Yuncheng Central Hospital between January 2024 and December 2025. General clinical data were collected, along with results for blood tests, fasting glucose levels, and lipid profiles from weeks 10~13 of pregnancy. The results of the glucose tolerance test conducted between 24 and 28 weeks of gestation were used to divide the study participants into two groups: a GDM group and a non-GDM group.

**Results:**

Compared with the non-GDM group, patients in the GDM group exhibited significantly elevated levels of the inflammatory markers NHR and UHR (*P* < 0.001). Glucose levels at 0 h, 1 h, and 2 h during the OGTT showed a gradual upward trend as NHR and UHR quartile groups increased (*P* < 0.001). Spearman’s rank correlation analysis revealed that serum NHR and UHR levels in women at 10~13 weeks’ gestation were positively correlated with GDM (*P* < 0.001). Multivariate logistic regression analysis indicated that both NHR and UHR were independent risk factors for the development of GDM (*P* < 0.001). ROC analysis demonstrated that NHR and UHR could serve as potential predictive markers for assessing the occurrence of GDM after 24 weeks of gestation.

**Conclusion:**

Elevated levels of NHR and UHR in early pregnancy are positively correlated with the development of GDM. They constitute independent risk factors for its onset and possess a certain degree of predictive value, thereby providing a reference for the early clinical prevention and management of GDM.

## 1 Introduction

Gestational diabetes mellitus (GDM) is a condition characterized by the first-time detection of glucose and lipid metabolism disorders during pregnancy; it is closely associated with adverse pregnancy outcomes and has a serious impact on the health of newborns (1). The global prevalence of GDM reaches 14% (2), and it has become a major risk factor for women developing type 2 diabetes mellitus (T2DM), whilst also significantly increasing their long-term risk of cardiovascular disease and diabetes (3). Compared with pregnant women with normal glucose tolerance, those with GDM face a higher risk of perinatal and obstetric complications, such as pre-eclampsia and cardiovascular metabolic disorders (4). Currently, clinical practice primarily relies on the oral glucose tolerance test (OGTT) conducted between 24 and 28 weeks of gestation to diagnose GDM (5). However, studies have shown that screening for and intervening in glucose metabolism abnormalities before 20 weeks of gestation can effectively reduce the incidence of adverse pregnancy outcomes (6). Therefore, the identification of GDM-specific biomarkers is of great significance for the precise identification of high-risk populations for GDM in early pregnancy.

Research reports indicate that chronic inflammation is associated with insulin resistance in patients with GDM, and serum levels of inflammatory cytokines can be used to predict its early onset (7). Another cohort study suggests that inflammatory cytokines may serve as biomarkers for GDM, implying that biological effects such as immune-endocrine interactions may play a central role in the onset and progression of gestational diabetes (8). In recent years, the neutrophil to high-density lipoprotein cholesterol ratio (NHR) and the uric acid to high-density lipoprotein cholesterol ratio (UHR) have emerged as novel inflammatory markers, playing a significant role in predicting conditions such as non-alcoholic fatty liver disease (NAFLD) (9), acute myocardial infarction (10), depression (11), and type 2 diabetes (12). In studies on GDM, Feng et al. (13) utilized data from the National Health and Nutrition Examination Survey (NHANES) to find a linear positive correlation between UHR and GDM, although its independent diagnostic capacity was limited. Meanwhile, Ma et al. (14) found that NHR levels were significantly elevated in the first trimester among GDM subjects and were closely associated with the onset of GDM.

Although the aforementioned studies indicate an association between NHR, UHR, and the incidence of GDM, there is currently a relative lack of research into the predictive value of NHR and UHR for GDM in early pregnancy, and no studies have yet examined the combined predictive value of these two factors for GDM. Therefore, this study aims to investigate the relationship between NHR and UHR in early pregnancy and the combined predictive value of these two factors for GDM, with a view to providing a reference for the early clinical prevention and treatment of GDM.

## 2 Materials and methods

### 2.1 Study population and data collection

**Figure 1:**
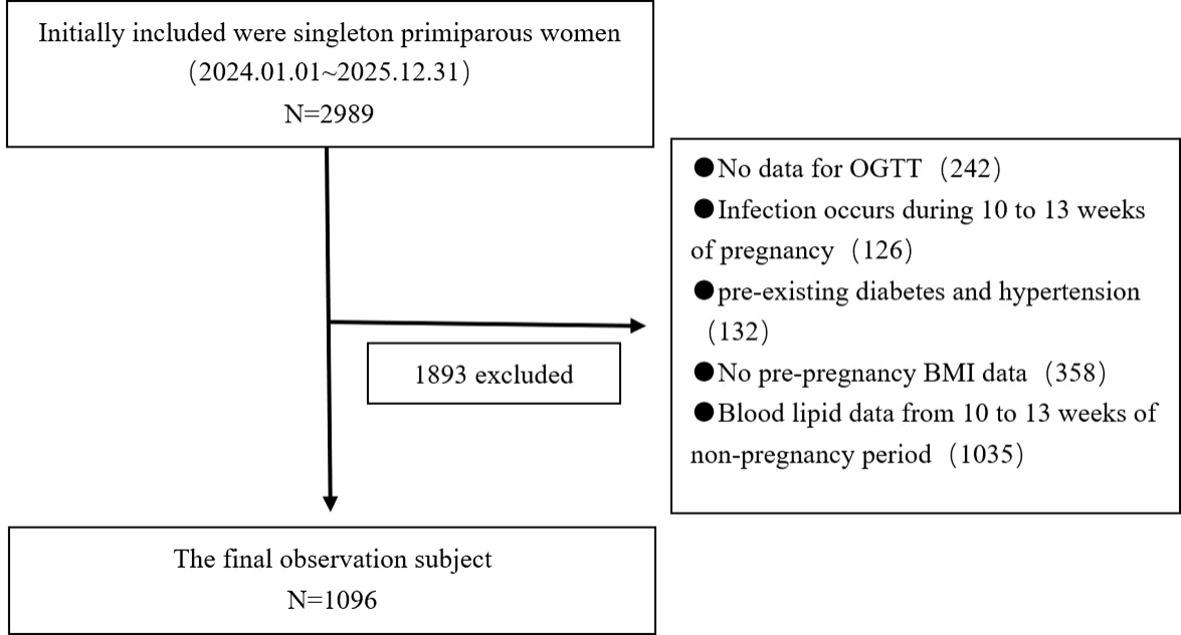
A flowchart of patients’ selection.

A retrospective analysis was conducted, selecting 2,989 singleton primiparous women who received routine antenatal care and examinations at the Obstetrics Department of Yuncheng Central Hospital between January 2024 and December 2025 as the study subjects. Clinical data were collected from the medical records system, including age, BMI, blood test results (complete blood count, fasting glucose, and lipid profile) obtained between 10 and 13 weeks of gestation. Inclusion criteria: age ≥ 18 years, singleton primiparous women, complete clinical records, and no other obstetric complications. Exclusion criteria: (1) pre-existing diabetes; (2) inflammatory conditions, such as respiratory or urinary tract infections; (3) comorbidities including hypertension, liver, kidney or pancreatic diseases, cardiovascular and cerebrovascular diseases, hematological disorders, autoimmune diseases, and malignancies. A total of 1,096 cases with complete data were included (**Figure 1**). Based on the results of the oral glucose tolerance test (OGTT) conducted between 24 and 28 weeks of gestation as recorded in the medical records system, the study subjects were divided into a GDM group (414 cases) and a non-GDM group (682 cases). Diagnostic criteria for GDM: Based on the results of the oral glucose tolerance test, the upper limits for fasting, 1-hour and 2-hour postprandial blood glucose levels are 5.1, 10.0 and 8.5 mmol/L, respectively; a diagnosis of GDM is made if blood glucose levels reach or exceed any of these thresholds (15).

As this study was a retrospective analysis, informed consent from participants could not be obtained. This study was approved by the ethics committee of our hospital, and its protocol abided by the principles of the Declaration of Helsinki (NO. YXLL-KS2026026).

### 2.2 Detection of blood markers and calculation of the inflammatory index

WBC (including neutrophil count) was measured using a standard automated hematology analyzer (SYSMEX-XN9000, Japan), with all reagents supplied by the manufacturer. Triglycerides (GPO-PAP method), total cholesterol (CHOD-PAP method), high-density lipoprotein cholesterol (direct method – peroxidase clearance method), low-density lipoprotein cholesterol (direct method – peroxidase clearance method), uric acid (uricase method) and blood glucose (hexokinase method) were measured using a fully automated biochemistry analyzer (Roche C701, Germany). The reagents for all the biochemical indicator tests were purchased from Roche Diagnostics Products (Shanghai) Co., Ltd. (Shanghai, China). The daily laboratory quality control for all these tests was within the acceptable range.

NHR = Neutrophil count(×10^9^/L)/HDL-C (mmol/L) ratio; UHR = SUA (umol/L)/HDL-C(mmol/L) ratio.

### 2.3 Statistical analysis

Statistical analysis was performed using SPSS 22.0 software. Non-normally distributed data are presented as the median and interquartile range [M (Q1, Q3)], whilst categorical variables are expressed as percentages. Comparisons of non-normally distributed quantitative variables between the two groups were performed using two-independent-samples nonparametric tests. Comparisons of non-normally distributed data across multiple groups were performed using non-parametric tests. Comparisons of rates for categorical variables were performed using the chi-square test. The correlation between NHR, UHR and GDM was analyzed using Spearman’s rank correlation. As the outcome variable in this study (GDM) was a binary categorical variable, multivariate logistic regression was used to analyze its influencing factors. The predictive value of NHR and UHR for GDM was assessed using ROC curves.

## 3 Results

### 3.1 Comparison of clinical characteristics and indicators between the GDM group and the non-GDM group

A total of 1096 pregnant women were included, among which 682 were in the non-GDM group and 414 were in the GDM group. There were no significant differences in factors such as age, blood pressure, WBC, LDL, TC and glucose between the two groups (*P* > 0.05). Compared with the non-GDM group, the pre-pregnancy BMI value of the GDM group was higher, and the levels of NEUT#, TG, Non-HDL-C and blood uric acid in the blood at 10~13 weeks of pregnancy were higher, while the HDL level was significantly lower (*P<*0.001). In the glucose tolerance test of the GDM group, the blood glucose levels at each time point were higher than those of the non-GDM group (*P<*0.001), and the NHR and UHR levels were also significantly increased (*P<*0.001). As shown in **Table 1**.

**Table 1:** Comparison of clinical characteristics and indicators between the GDM group and the non-GDM group [M (Q1, Q3)].

Characteristics	non-GDM (n=682)	GDM (n=414)	*χ^2^/Z*	*P*
Age (years)	30 (28,32)	30 (28,33)	-1.484	0.138
Pre-pregnancy BMI (kg/m²)	21.33 (20.17,22.78)	24.89 (21.98,26.77)	-15.493	0.000
WBC	5.89 (5.34,6.78)	6.11 (4.91,7.25)	-0.292	0.770
NEUT # (×10^9^/L)	3.84 (3.11,4.35)	4.10 (3.50,5.11)	-6.585	0.000
LDL-C (mmol/L)	2.36 (2.01,3.00)	2.56 (1.92,3.34)	-1.600	0.110
HDL-C (mmol/L)	1.23 (1.04,1.47)	1.02 (0.84,1.17)	-12.764	0.000
TG (mmol/L)	1.25 (0.86,1.96)	1.66 (1.21,2.61)	-7.859	0.000
TC (mmol/L)	4.04 (3.67,4.90)	4.24 (3.40,5.34)	-0.813	0.416
Non-HDL-C (mmol/L)	2.79 (2.43,3.58)	3.21 (2.48,4.11)	-4.543	0.000
SUA (umol/L)	205.00 (179.00,237.00)	234.50 (203.00,274.00)	-10.489	0.000
Glucose (mmol/L)	4.73 (4.44,5.02)	4.80 (4.38,5.18)	-1.813	0.070
OGTT 0h (mmol/L)	4.42 (4.20,4.68)	5.00 (4.65,5.29)	-15.626	0.000
OGTT 1h (mmol/L)	7.13 (6.22,8.25)	9.93 (8.96,11.61)	-23.484	0.000
OGTT 2h (mmol/L)	6.25 (5.32,6.99)	8.15 (7.10,9.22)	-20.063	0.000
NHR	3.12 (2.38,3.86)	4.29 (3.04,5.38)	-13.101	0.000
UHR	165.16 (135.69,206.04)	240.57 (197.96,290.91)	-16.499	0.000

Data are expressed as n (%) for categorical variables and as M (Q1, Q3) for continuous variables with non-normal distribution. M (Q1, Q3), median (interquartile range); WBC, white blood cell; NEUT #, absolute neutrophil count; LDL-C, low-density lipoprotein cholesterol; HDL-C, high-density lipoprotein cholesterol; TG, triglyceride; TC, total cholesterol; non-high-density lipoprotein cholesterol; SUA, serum uric acid; Glucose: Serum glucose at 10~13 weeks of gestation; OGTT 0h, Fasting plasma glucose (FPG) in oral glucose tolerance test; OGTT 1h, 1-hour plasma glucose in oral glucose tolerance test; OGTT 2h, 2-hour plasma glucose in oral glucose tolerance test; NHR, neutrophil to high-density lipoprotein ratio; UHR, uric acid to high-density lipoprotein cholesterol ratio. *P* < 0.05 was considered statistically significant.

### 3.2 The relationship between NHR, UHR and GDM

#### 3.2.1 The results of the OGTT classified according to the NHR and UHR quartiles

The study participants were divided into four groups based on the quartiles of NHR and UHR, and the differences in blood glucose levels at various time points during the OGTT were compared across the groups. The results showed that blood glucose levels at 0 h, 1 h and 2 h during the OGTT increased progressively with rising NHR and UHR quartiles (*P<*0.001). This suggests that women in the higher quartiles of NHR or UHR have a higher incidence of GDM. As shown in **Table 2**.

**Table 2:** The results of the oral glucose tolerance test (OGTT) classified according to the NHR and UHR quartiles [M (Q1, Q3)].

Characteristics		OGTT 0h (mmol/L)	OGTT 1h (mmol/L)	OGTT 2h (mmol/L)
NHR	Q1 (<2.61)	4.40 (4.20,4.74)	7.19 (6.27,8.86)	6.25 (5.37,7.15)
	Q2 (2.61~)	4.50 (4.22,4.79)	7.55 (6.33,8.90)	6.47 (5.47,7.23)
	Q3 (3.40~)	4.59 (4.27,4.90)	8.23 (6.95,9.38)	6.60 (5.73,7.73)
	Q4 (≥4.41)	4.92 (4.45,5.27)	9.52 (8.28,10.87)	7.85 (6.42,9.00)
	*Z*	81.094	130.023	118.481
	*P*	0.000	0.000	0.000
UHR	Q1 (<147.40)	4.40 (4.17,4.67)	7.09 (6.10,8.34)	6.21 (5.10,7.01)
	Q2 (147.40~)	4.54 (4.24,4.85)	7.65 (6.41,8.94)	6.49 (5.56,7.25)
	Q3 (190.81~)	4.59 (4.22,5.03)	8.31 (6.72,9.88)	6.68 (5.62,8.22)
	Q4 (≥243.75)	4.95 (4.55,5.27)	9.71 (8.54,11.09)	7.91 (6.60,9.06)
	*Z*	128.684	206.432	168.945
	*P*	0.000	0.000	0.000

Data are expressed as n (%) for categorical variables and as M (Q1, Q3) for continuous variables with non-normal distribution. M (Q1, Q3), median (interquartile range); OGTT 0h, Fasting plasma glucose (FPG) in oral glucose tolerance test; OGTT 1h, 1-hour plasma glucose in oral glucose tolerance test; OGTT 2h, 2-hour plasma glucose in oral glucose tolerance test; NHR, neutrophil to-high-density lipoprotein ratio; UHR, uric acid to high-density lipoprotein cholesterol ratio. *P* < 0.05 was considered statistically significant.

#### 3.2.2 The correlation between NHR and UHR levels and GDM

The results of the correlation analysis between serum NHR and UHR levels in women at 10~13 weeks of gestation and GDM are shown in **Table 3**. NHR and UHR were positively correlated with GDM (*P<*0.001). The rank correlation coefficients between NHR and blood glucose levels at each time point of the OGTT (meeting the criteria for GDM diagnosis) were 0.271, 0.357 and 0.333, respectively; the rank correlation coefficients between UHR and blood glucose levels at each time point of the OGTT (meeting the criteria for GDM diagnosis) were 0.329, 0.432 and 0.389, respectively.

**Table 3:** The correlation between NHR and UHR levels and GDM.

Characteristics	NHR		UHR	
	*r_s_*	*P*	*r_s_*	*P*
OGTT 0h (>5.1 mmol/L)	0.271	0.000	0.329	0.000
OGTT 1h (>10.0 mmol/L)	0.357	0.000	0.432	0.000
OGTT 2h (>8.5 mmol/L)	0.333	0.000	0.389	0.000

Data are expressed as n (%) for categorical variables and as M (Q1, Q3) for continuous variables with non-normal distribution. M (Q1, Q3), median (interquartile range); OGTT 0h: Fasting plasma glucose (FPG) in oral glucose tolerance test; OGTT 1h: 1-hour plasma glucose in oral glucose tolerance test; OGTT 2h: 2-hour plasma glucose in oral glucose tolerance test; NHR, neutrophil to high-density lipoprotein ratio; UHR, uric acid to high-density lipoprotein cholesterol ratio. *P* < 0.05 was considered statistically significant.

### 3.3 Multivariate logistic regression analysis

The risk factors related to GDM that showed statistically significant differences in the single-factor analysis were selected as independent variables, and the occurrence of GDM (represented by 1 for “yes” and 0 for “no”) was taken as the dependent variable for the multivariate logistic regression analysis. Multicollinearity of all variables in the regression model was evaluated by variance inflation factor (VIF). A VIF value < 5 was defined as the absence of significant multicollinearity. In this study, BMI, TG, NHR and UHR were simultaneously included in the predictive model; meanwhile, their original constituent indicators (neutrophil count, uric acid, HDL-C) and other lipid-related indicators were not enrolled synchronously, so as to avoid obvious collinearity between raw parameters and derived ratio indicators. The results showed that both NHR and UHR levels in the blood of women at 10~13 weeks’ gestation were independent risk factors for the development of GDM (*P<*0.001). As shown in **Table 4**.

**Table 4:** Multivariate logistic regression analysis for GDM.

Indicators	β	Standard error	Wald χ^2^	Odds ratio	95% CI	*P*
Pre-pregnancy BMI	0.422	0.035	147.417	1.525	1.424-1.632	0.000
TG	0.357	0.077	2.863	0.987	0.071-1.043	0.026
NHR	0.471	0.072	42.171	1.601	1.389-1.846	0.000
UHR	0.013	0.002	70.448	1.013	1.010-1.016	0.000

NHR, neutrophil to high-density lipoprotein ratio; UHR, uric acid to high-density lipoprotein cholesterol ratio. *P* < 0.05 was considered statistically significant. *P* < 0.05 was considered statistically significant.

### 3.4 ROC curve analysis

**Figure 2:**
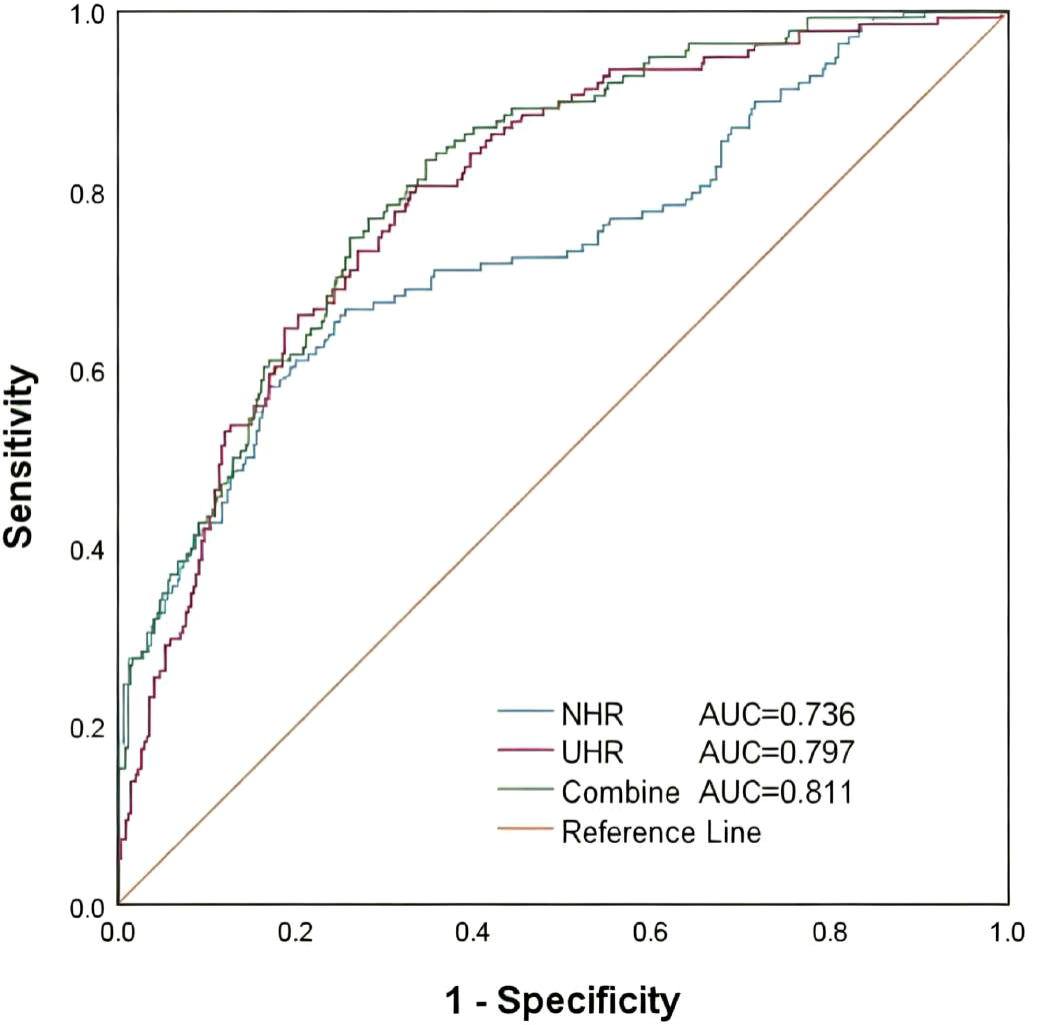
ROC curve analysis for GDM. AUC, area under the curve; NHR, neutrophil to-high-density lipoprotein ratio; UHR, uric acid to high-density lipoprotein cholesterol ratio.

This study employed ROC curves to evaluate the predictive performance of NHR and UHR for GDM. The results showed that the AUC values for NHR and UHR were 0.736 and 0.797, respectively (*P* < 0.001); when tested in combination, the AUC increased to 0.811, with sensitivity and specificity reaching 80.23% and 76.29%, respectively. This indicates that the combined detection of NHR and UHR may serve as a potential predictive indicator for assessing the occurrence of GDM after 24 weeks of gestation. As shown in **Table 5** and **Figure 2**.

**Table 5:** ROC curve analysis for GDM.

Indicators	Sensitivity (%)	Specificity (%)	Cut-off point	AUC	95% CI	*P*
NHR	71.51	68.23	3.25	0.736	0.704-0.767	0.000
UHR	76.79	72.54	185.62	0.797	0.770-0.823	0.000
Combine	80.23	76.29	0.418	0.811	0.786-0.837	0.000

AUC, Area under the curve; NHR, Neutrophil to-high-density lipoprotein ratio; UHR, urate-to-high-density lipoprotein cholesterol ratio. *P* < 0.05 was considered statistically significant.

## 4 Discussion

GDM is a specific form of diabetes diagnosed for the first time during pregnancy in women who had normal glucose metabolism or impaired glucose tolerance prior to conception. The current diagnostic criteria for GDM are primarily based on the 2010 recommendations of the International Association of Diabetes and Pregnancy Study Group (IADPSG) (16), which involve conducting an OGTT during the second trimester (24–28 weeks of gestation). This method involves a relatively late diagnosis and lacks indicators for early detection. Currently, interventions for GDM primarily involve nutritional management and exercise control; the earlier the intervention, the easier it is to mitigate the risks associated with GDM, making early prediction of GDM essential. Research has indicated (17)that GDM is associated with a state of chronic low-grade inflammation in the pregnant woman. Throughout pregnancy, a balance between pro-inflammatory and anti-inflammatory cytokines is required to regulate the maternal inflammatory system and achieve a state of dynamic stability. Furthermore, studies have suggested that lipid metabolism in women with GDM is likely to have already undergone changes prior to pregnancy, although these are not detectable in routine examinations (18). The results of this study indicate that, compared with pregnant women without GDM, there were no significant differences in levels of WBC, TC and fasting blood glucose in the early stages of pregnancy among women with GDM. However, levels of pre-pregnancy BMI, neutrophils, TG, non-HDL-C, serum uric acid, non-fasting renal excretion of NHR and UHR were elevated, whilst HDL levels were significantly reduced. This is consistent with the findings of the aforementioned studies (17, 18). This suggests that the bodies of women with GDM may be in a state of chronic, low-grade inflammation during early pregnancy; if this condition is not corrected in a timely manner, it may contribute to the development of GDM.

The inflammatory profile of pregnancy differs from that of the non-pregnant state; normal pregnancy is characterized by pro-inflammatory and pro-thrombotic states, as well as high insulin resistance and hyperlipidemia (19). An imbalance between pro-inflammatory and anti-inflammatory factors may be a key cause of various complications during pregnancy (20). Within the body, metabolic regulation and immune responses are highly integrated (21). Multiple inflammatory factors are involved in regulating various metabolic products, such as lipids and glucose, whilst intense inflammation may lead to the excessive accumulation of abnormal levels of lipids and glucose, ultimately resulting in metabolic dysfunction and a loss of homeostasis (22). Therefore, it is of great significance to investigate the biological roles of inflammatory and metabolic markers in the onset and progression of GDM.

Neutrophils are one of the major subsets of white blood cells and have been shown to play a significant role in the body’s inflammatory response (23). When inflammation occurs, they are the first immune cells to respond, assisting in the recruitment of macrophages whilst interacting with antigen-presenting cells, thereby further promoting chronic inflammatory responses (24). Uric acid is synthesized by xanthine oxidase during purine metabolism and acts as an extracellular antioxidant, helping to prevent oxidative stress. Normal uric acid levels exert an antioxidant effect, whereas elevated levels promote oxidative stress (25). When serum uric acid rises during pregnancy, it disrupts glucose homeostasis via pathways such as inflammation, oxidative stress and endothelial damage, thereby contributing to the pathogenesis of GDM (26, 27). HDL-C, as a typical lipid-related biomarker, can remove excess cholesterol from peripheral tissues; furthermore, it inhibits the expression of endothelial cell adhesion molecules, thereby preventing the accumulation of monocytes on the arterial wall (28, 29). Concurrently, HDL-C plays a significant role in the activation, migration and diffusion of neutrophils (30). Consequently, HDL-C exerts important anti-inflammatory and antioxidant protective effects (31).Although there were significant differences in the levels of neutrophils, serum uric acid and HDL-C between the two groups of pregnant women in this study during early pregnancy, most of these indicators fell within the normal reference range and were therefore unable to serve as effective warning signs. Consequently, novel inflammatory markers based on blood cell subsets and biochemical indicators (such as HDL-C and serum uric acid) have emerged, providing medical researchers with new and more comprehensive avenues for investigation.

NHR is a novel, comprehensive inflammatory-metabolic marker derived from neutrophils and HDL-C, capable of providing a more objective reflection of changes in the body’s inflammation and oxidative stress (32). UHR, on the other hand, is the product of uric acid and HDL-C and serves as a marker of increased inflammatory response in the body, as well as a new inflammatory and metabolic marker (12). The results of this study indicate that patients with GDM exhibit higher levels of NHR and UHR during the first trimester of pregnancy. Furthermore, in the OGTT, the overall blood glucose levels at 0, 1 and 2 hours showed that as NHR and UHR increased from the first quartile to the fourth quartile, overall blood glucose levels also increased. This suggests that women with NHR or UHR in the higher quartiles during early pregnancy have a higher probability of developing GDM. Furthermore, results from multivariate ordered regression analysis showed that both NHR and UHR are independent risk factors for the development of GDM. Among these, NHR demonstrated a stronger association with GDM (OR = 1.523, 95% CI: 1.421–1.633, *P* < 0.001), suggesting that it may be a biomarker of greater clinical value; UHR also demonstrated a significant positive association (OR = 1.250, 95% CI: 1.032–1.514, *P* = 0.023), further supporting the important role of inflammation and dyslipidemia in the pathophysiological process of GDM. Furthermore, ROC analysis demonstrated that NHR and UHR in early pregnancy possess good diagnostic performance for the prediction of GDM, with combined testing yielding higher diagnostic performance. This suggests that NHR and UHR can serve as clinical indicators for predicting the onset of GDM in early pregnancy, with combined testing yielding better results.

Our study indicates that elevated levels of NHR and UHR in early pregnancy are positively correlated with the development of GDM and constitute independent risk factors for its onset. The potential biological mechanisms underlying the involvement of these two markers in the development of GDM may be related to the following: (1) insulin resistance. Ultimately, GDM remains a form of diabetes, and insulin resistance is a hallmark feature of the disease. GDM is characterized by impaired glucose tolerance resulting from dysfunction of the maternal pancreatic β-cells, which in turn leads to insufficient insulin production to regulate glucose homeostasis during pregnancy (33). During normal pregnancy, various hormones secreted by the placenta, such as estrogen and progesterone, combined with elevated maternal cortisol levels, collectively contribute to physiological insulin resistance (34, 35). Neutrophil levels in pregnant women are significantly higher than before pregnancy, and increased neutrophil activity leads to an increase in reactive oxygen species (ROS). These ROS can induce downstream inflammatory responses and insulin resistance by generating neutrophil extracellular traps (36, 37). In early pregnancy, serum uric acid not only impairs vascular endothelial function by inhibiting endothelial nitric oxide synthase activity and reducing nitric oxide bioavailability but also acts as a pro-oxidant to activate the oxidase system (38). This induces the production of large amounts of ROS and triggers metabolic inflammation. These combined pathological changes collectively exacerbate insulin resistance during pregnancy, thereby promoting the progression of gestational diabetes. (2) Inflammatory response. Pregnancy represents a unique phase of immune regulation in women. During this period, the interplay between pro-inflammatory and anti-inflammatory factors is crucial for maintaining the dynamic immune balance in pregnant women. However, this balance is disrupted when pro-inflammatory cytokines, such as neutrophils and C-reactive protein, are abnormally elevated, leading to chronic low-grade inflammation (39). These pro-inflammatory factors can stimulate endoplasmic reticulum stress and oxidative stress, directly impairing β-cell function (40), which in turn leads to impaired glucose tolerance in pregnant women. Serum uric acid also possesses oxidizing properties; elevated levels can cause glucose metabolism disorders via pathways such as endothelial cell damage and inflammation (41). Persistent hyperglycemia exacerbates inflammatory responses and the imbalance in oxidative stress, creating a vicious cycle that drives the development of GDM. (3) Abnormal lipid metabolism. Numerous previous studies have demonstrated a strong association between maternal dyslipidemia and GDM. For example, Hu et al. (42) reported that TG levels in women with GDM were on average 20% higher than in healthy pregnant women; elevated TG levels increase free fatty acid (FFA) levels via lipolysis, thereby exacerbating and accelerating the onset of GDM. The findings of Wang et al. (43) indicate that elevated levels of residual cholesterol are associated with an increased risk of gestational diabetes; this may be due to its pro-inflammatory properties exacerbating inflammatory responses and interfering with the physiological function of glucose kinase, thereby promoting the development of insulin resistance. Furthermore, HDL-C, often referred to as the “only good cholesterol”, exerts anti-inflammatory and antioxidant effects by scavenging oxidized lipids. Concurrently, it mediates reverse cholesterol transport, removing excess cholesterol from peripheral tissues and thereby maintaining lipid homeostasis. When HDL-C levels decline or its function is impaired, these protective functions are diminished (44). Persistent lipid metabolism abnormalities can lead to abnormal lipid deposition in pancreatic β-cells, resulting in impaired insulin secretion. Consequently, NHR and UHR may serve as direct indicators of the involvement of factors such as neutrophils, uric acid and HDL-C in the body’s chronic inflammatory response.

To our knowledge, this is the first study to combine NHR and UHR in the field of GDM. Neutrophils, serum uric acid and HDL-C are all routine laboratory parameters characterized by low cost and ease of testing, making them suitable for initial screening in primary care settings. As a combination of these three markers, which covers different metabolic dimensions such as “inflammatory response and stress” and “lipid metabolism”, NHR and UHR hold broad prospects for application in GDM. However, neutrophils themselves are influenced by a variety of diseases and lack specificity. Uric acid is affected by multiple factors including diet, renal function and medication, whilst HDL-C is regulated by genetics, exercise and diet. These factors may all reduce the specificity of NHR and UHR for GDM. Consequently, it must be acknowledged that this constitutes a limitation of the present study. Furthermore, confounding factors such as family history of diabetes, diet and physical activity were not included in this study, as data on these aspects were missing from the majority of medical records. This might have limited the persuasiveness of the results of this study. Furthermore, since there may be significant differences in lipid metabolism and insulin resistance among different ethnic groups (45), the conclusions of this study have limitations when applied across different ethnic groups. The retrospective nature of the study also limits the persuasiveness of the findings, as it does not allow for the comprehensive observation of dynamic changes in these indicators over time, unlike long-term follow-up studies. Finally, the AUC values for NHR, UHR, and combined detection were 0.736, 0.797, and 0.811 respectively. The results were overly optimistic, and this might be related to the fact that lipid checks were not conducted in the early stages of pregnancy in clinical practice. A prospective study might be able to address this issue. In the future, we will also conduct prospective research to enhance its persuasiveness. In addition, we also observed that some studies have emphasized that subclinical myocardial dysfunction, particularly impaired global longitudinal strain, may already be present in women with GDM even before overt cardiac abnormalities are detectable (46, 47). Therefore, in the future, we will also use indicators such as NHR and UHR to explore the relationship between inflammation and metabolic changes and myocardial strain parameters in GDM women, which could provide novel insights into early cardiovascular risk stratification and help identify patients who may benefit from closer monitoring and targeted interventions.

## Conclusion

NHR and UHR are highly valuable inflammatory indicators. In our study, the results showed that the increase of NHR and UHR in the early pregnancy was positively correlated with the occurrence of GDM. They are independent risk factors for its onset and have certain predictive value, which may provide a reference for the early clinical prevention and management of GDM.

## Data Availability

The raw data supporting the conclusions of this article will be made available by the authors, without undue reservation.
